# Supplementary material for: Polyketide synthase gene clusters in the ericoid mycorrhizal fungus Oidiodendron maius: genome-wide prediction, expression profiling, and functional characterization of knockout mutants
Source: Front Plant Sci. 2026 Sep 16;17:1924430. doi: 10.3389/fpls.2026.1924430 (PMC13623903; doi:10.3389/fpls.2026.1924430)
Supplement: Supplementary file 1 [file SupplementaryFile1.zip › Supplementary Materials/Supplementary Folder 1.zip!/Oidma1_AssemblyScaffolds_Repeatmasked/knownclusterblast/cluster30/jgi.p_Oidma1_44051_mibig_hits.html]

| MIBiG Protein | Description | MIBiG Cluster | MiBiG Product | % ID | % Coverage | BLAST Score | E-value |
| --- | --- | --- | --- | --- | --- | --- | --- |
| EPE34336.1 | Cytochrome\_P450 | BGC0001035 | Pneumocandin\_biosynthetic\_gene\_cluster | 32 | 93.75 | 246 | 6.2e-65 |
| AFT91399.1 | HtyF | BGC0000338\_c2 | Echinocandin\_B\_biosynthetic\_gene\_cluster | 34 | 83.3333333333 | 212 | 1.3e-54 |
| AMM63168.1 | AniF2 | BGC0001371 | Echinocandin\_B\_biosynthetic\_gene\_cluster | 34 | 87.6893939394 | 206 | 7.1e-53 |
| CBX55165.1 | P450\_monooxygenase | BGC0001243 | Botryenalol\_/\_botrydial\_/\_dihydrobotrydial\_/\_botryendial\_/ | 30 | 90.9090909091 | 180 | 5.4e-45 |
| EAU29532.1 | conserved\_hypothetical\_protein | BGC0000682 | Terretonin\_biosynthetic\_gene\_cluster | 31 | 75.7575757576 | 169 | 1.3e-41 |
| BAM84048.1 | cytochrome\_P450\_monooxygenase | BGC0001260 | Terpendole\_E\_biosynthetic\_gene\_cluster | 31 | 66.2878787879 | 168 | 1.6e-41 |
| XP\_002384045.1 | ferulate-5-hydroxylase,\_putative | BGC0001234 | 4,4'-piperazine-2,5-diyldimethyl-bis-phenol\_biosynthetic\_gene | 30 | 83.7121212121 | 167 | 3.7e-41 |
| BAD29974.1 | P450\_monooxygenase\_2 | BGC0000676 | Aphidicolin\_biosynthetic\_gene\_cluster | 28 | 89.0151515152 | 166 | 6.2e-41 |
| BAE56598.1 |  | BGC0001123 | Aspirochlorine\_biosynthetic\_gene\_cluster | 29 | 86.1742424242 | 157 | 4.9e-38 |
| EAU36745.1 | hypothetical\_protein | BGC0000292 | Acetylaranotin\_biosynthetic\_gene\_cluster | 27 | 85.2272727273 | 152 | 1.6e-36 |
| CBF87243.1 | cytochrome\_P450,\_putative\_(Eurofung) | BGC0000680\_c2 | Austinol/dehydroaustinol,\_locus\_1\_biosynthetic\_gene\_cluster | 29 | 84.6590909091 | 151 | 2.7e-36 |
| CAA75566.1 | cytochrome\_P450\_monooxygenase | BGC0001270\_c2 | Gibberellin\_A3\_/\_gibberellin\_A1\_/\_gibberellin\_A7\_/\_gibberellin\_A4\_/ | 25 | 88.8257575758 | 149 | 7.9e-36 |
| EBA27371.1 | cytochrome\_P450\_monooxygenase,\_putative | BGC0000129 | Pyripyropene\_biosynthetic\_gene\_cluster | 29 | 74.053030303 | 142 | 1.3e-33 |
| CBF87249.1 | cytochrome\_P450,\_putative\_(Eurofung) | BGC0000680\_c2 | Austinol/dehydroaustinol,\_locus\_1\_biosynthetic\_gene\_cluster | 27 | 96.5909090909 | 130 | 4.9e-30 |
| CAP07651.1 | P450\_monooxygenase | BGC0001269\_c5 | Gibberellin\_A3\_/\_gibberellin\_A1\_/\_gibberellin\_A7\_/\_gibberellin\_A4\_/ | 27 | 75.1893939394 | 130 | 6.5e-30 |
| AGA37282.1 | P450\_monooxygenase | BGC0000819 | Paraherquamide\_biosynthetic\_gene\_cluster | 30 | 65.5303030303 | 129 | 1.1e-29 |
| CAP07653.1 | P450\_monooxygenase | BGC0001269\_c3 | Gibberellin\_A3\_/\_gibberellin\_A1\_/\_gibberellin\_A7\_/\_gibberellin\_A4\_/ | 24 | 94.5075757576 | 124 | 4.6e-28 |
| BAV32161.1 | cytochrome\_P450\_monooxygenase | BGC0001373 | Sordarin\_biosynthetic\_gene\_cluster | 26 | 67.803030303 | 122 | 1.8e-27 |
| BAC20565.1 | cytochrome\_P450 | BGC0000039 | Compactin\_biosynthetic\_gene\_cluster | 26 | 64.2045454545 | 109 | 1.2e-23 |
| ctg1\_orf003 |  | BGC0001068 | Pyripyropene\_A\_biosynthetic\_gene\_cluster | 34 | 27.6515151515 | 82 | 1.2e-15 |
| ctg1\_orf00001 |  | BGC0000685 | Brassicicene\_C\_biosynthetic\_gene\_cluster | 38 | 20.4545454545 | 68 | 2.3e-11 |
| ctg1\_orf5 |  | BGC0000321 | Chaetocin\_biosynthetic\_gene\_cluster | 24 | 51.5151515152 | 68 | 2.3e-11 |
